# No correlation between radiolucency and biomechanical stability of keeled and pegged glenoid components

**DOI:** 10.1186/s12891-017-1550-0

**Published:** 2017-05-25

**Authors:** Andreas Voss, Knut Beitzel, Elifho Obopilwe, Stefan Buchmann, John Apostolakos, Jessica Di Venere, Michael Nowak, Mark P. Cote, Anthony A. Romeo, Augustus D. Mazzocca

**Affiliations:** 1 Department of Orthopaedic Surgery, University of Connecticut, Farmington, CT USA; 2 Department of Orthopaedic Sports Medicine, Technical University, Munich, Germany; 3 Orthopaedisches Fachzentrum Weilheim-Garmisch-Starnberg-Penzberg, Weilheim, Germany; 4 Department of Orthopaedic Surgery, Rush University Medical Center, Chicago, IL USA; 5 Department of Civil, Environmental and Biomedical Engineering, College of Engineering, Technology and Architecture, University of Hartford, West Hartford, CT USA

**Keywords:** Basic science study, Biomechanics, Keel glenoid, Peg glenoid, Shoulder prosthesis, Biomechanics

## Abstract

**Background:**

The purpose of this study was to examine biomechanical properties and the degree of radiolucency of two cemented basic glenoid designs for total shoulder arthroplasty. Our hypothesis was that a component with increased micro-motion in the laboratory at time zero would also exhibit a greater amount of radiolucency in patients at a minimum of 2 years post total shoulder arthroplasty.

**Methods:**

Thirty cadaveric shoulders were divided into 2 groups (keel vs. peg). The glenoid components were first loaded with a single axial eccentric force of 196 N in all orientations and then with a transversal load of 49 N to simulate in vivo loads with abduction. Displacement of the glenoid component was determined with four different linear variable-differential transducers. In the second phase, 56 antero-posterior x-rays of 52 patients with either the same keeled (*n* = 24) or pegged (*n* = 32) glenoid component with a minimum of 24 months follow-up were evaluated for radiolucency.

**Results:**

Biomechanically the pegged glenoid showed a significant increase in micro-motion during eccentric axial loading as well as during combined loading in the anterior, posterior, and inferior position as compared to the keeled glenoid (*p* < 0.05). In contrast all results were significant with greater radiolucency for the keeled glenoid component (*p* = 0.001).

**Conclusion:**

While the pegged component exhibited a greater amount of micro-motion during biomechanical testing, radiolucency was greater in patients with a keeled component. These findings provide support for both components from different perspectives and highlight the need for well-constructed studies to determine whether glenoid design has an effect on clinical outcome, because influences are multifactorial and biomechanical forces may not recreate forces seen in vivo.

## Background

Glenoid component loosening is still one of the major problems in shoulder arthroplasty. According to a systematic review of the current literature, radiolucent lines have been reported to occur at a rate of 7.3% per year with over 70% prevalence at 10 years follow up of total shoulder arthroplasties. Revisions due to glenoid loosening were performed at close to 1% per year following implantation [1]. Multiple factors including the method of glenoid preparation, cementing technique, implant-material etc. are considered potential reasons for loosening within the cement-bone interface. In the systematic review, the significant factors included Walch classification, gender, and diagnosis [1]. The design of the glenoid component (implant) has been suggested as another critical factor and therefore has led to concerns regarding optimal prosthetic design.

Adequate initial fixation strength is thought to be crucial for long-term stability of the glenoid component and ultimately the clinical success of total shoulder arthroplasty. Previous authors have identified eccentric loading and the resulting rocking of the glenoid component as an important biomechanical factor for implant loosening [2–4]. As a result, many designs have been developed with the intent to improve fixation of the glenoid. To date, keeled and pegged constructions have emerged as the most widely utilized designs. Results of recent radiographic studies have favored the pegged over the keeled glenoid designs at early follow-up such as 26 months [5]. However, others could not demonstrate significant differences in radiographic follow up studies with an intermediate follow up (<45 months) [6].

Our objective was to evaluate the effects of implant design (keel vs. peg) on initial stability and postoperative radiolucency. The purpose of this study was to determine whether the morphology of the glenoid that yielded the strongest primary stability with the least amount of micro-motion under eccentric loading would also exhibit less radiolucency in patients at a minimum of 2 years following total shoulder arthroplasty.

## Methods

### Part 1: biomechanics

#### Specimens

Thirty fresh-frozen (12 paired, 18 unpaired) cadaveric shoulders were used in this study. The 18 un-paired shoulders were randomly distributed into one of two groups. The paired shoulders were evenly distributed between the two groups. All shoulder specimens were thawed overnight at room temperature. Each shoulder was inspected for degenerative changes due to glenohumeral arthritis (shoulders demonstrating significant posterior glenoid wear were excluded), disarticulated at the glenohumeral joint, and the scapula was dissected free of all soft tissue. All specimens then underwent bone density evaluation with a dual-energy x-ray absorptiometry scan (Lunar DPX IQ; GE Healthcare, Chicago, IL, USA ). The scapula was then potted using plaster of Paris.

#### Implantation of glenoid component

The aim of the study was to compare two basic types of glenoid designs and their influence on micro instability. Therefore, pegged and keeled glenoid components produced by one company (Arthrex Inc., Naples, FL, USA) were used for this study.

**Fig. 1:**
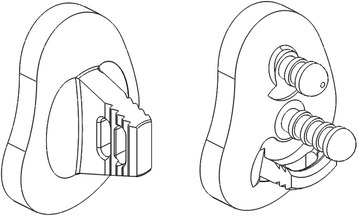
Keeled (*left*) and a pegged (*right*) glenoid component (Arthrex Inc., Naples, FL, USA) combine fenestration to improve anchoring and reverse barbs for better expansion effect and fixation strength. The main difference between the glenoids is the way of anchoring. The keeled glenoid consists of one single keeled anchor witch two fenestrations, the pegged glenoid consists of two pegged anchors and a curved keeled at the inferior part of the glenoid

Both glenoid implants combine fenestration to improve anchoring and reverse barbs for better expansion effect and fixation strength. The main difference between the implants is the physical design. The keeled glenoid consists of one single keeled anchor with two fenestrations while the pegged glenoid consists of two pegged anchors and a curved keeled at the inferior part of the glenoid. It must be mentioned that the pegged design itself with reversed barbs and an inferior keel is unlike other pegged designs from different companies. This may have a significant implication for in vitro and in vivo results (Fig. 1).

Prior to biomechanical testing, appropriate cement technique was verified. A small, medium, or large pegged or keeled glenoid was selected according to the bony dimensions of the glenoid and the instructions provided in the surgical technique guide. To prepare the glenoid surface an appropriately sized reamer was used until the superior to inferior surface was leveled and congruent to the implant. A Glenoid Punch (Arthrex Inc., Naples, FL, USA) for the keeled or the pegged component was then utilized to finish preparation. The cement (Simplex P Bone Cement, Stryker, Kalamazii, MI, USA) volume and weight was measured both prior to and after insertion to ensure accuracy of measurements. The overlapping bone cement secondary to glenoid implantation was removed and measured. The difference between the original amount of cement and the amount removed was then defined as used cement volume. The volume of cement for each implant was determined to address the different bone anchoring designs (pegged vs. keeled).

#### Biomechanical testing

**Fig. 2:**
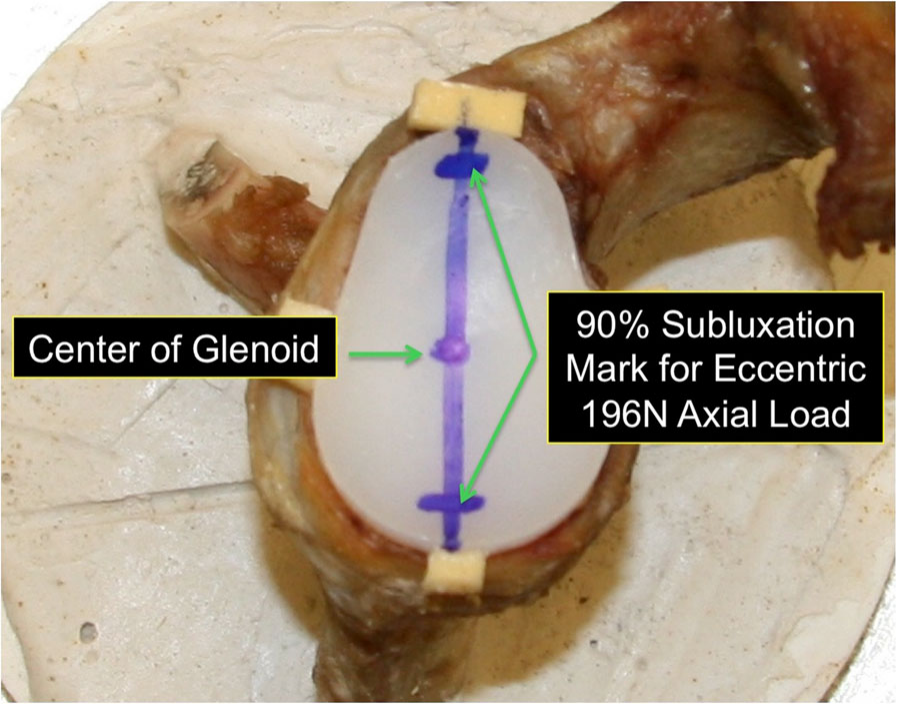
To define the eccentric point of loading a 90% subluxation point was selected on the glenoid in each direction. The figure shows the eccentric loading point for superior and inferior loading with a 196 N axial load and no transversal load

**Fig. 3:**
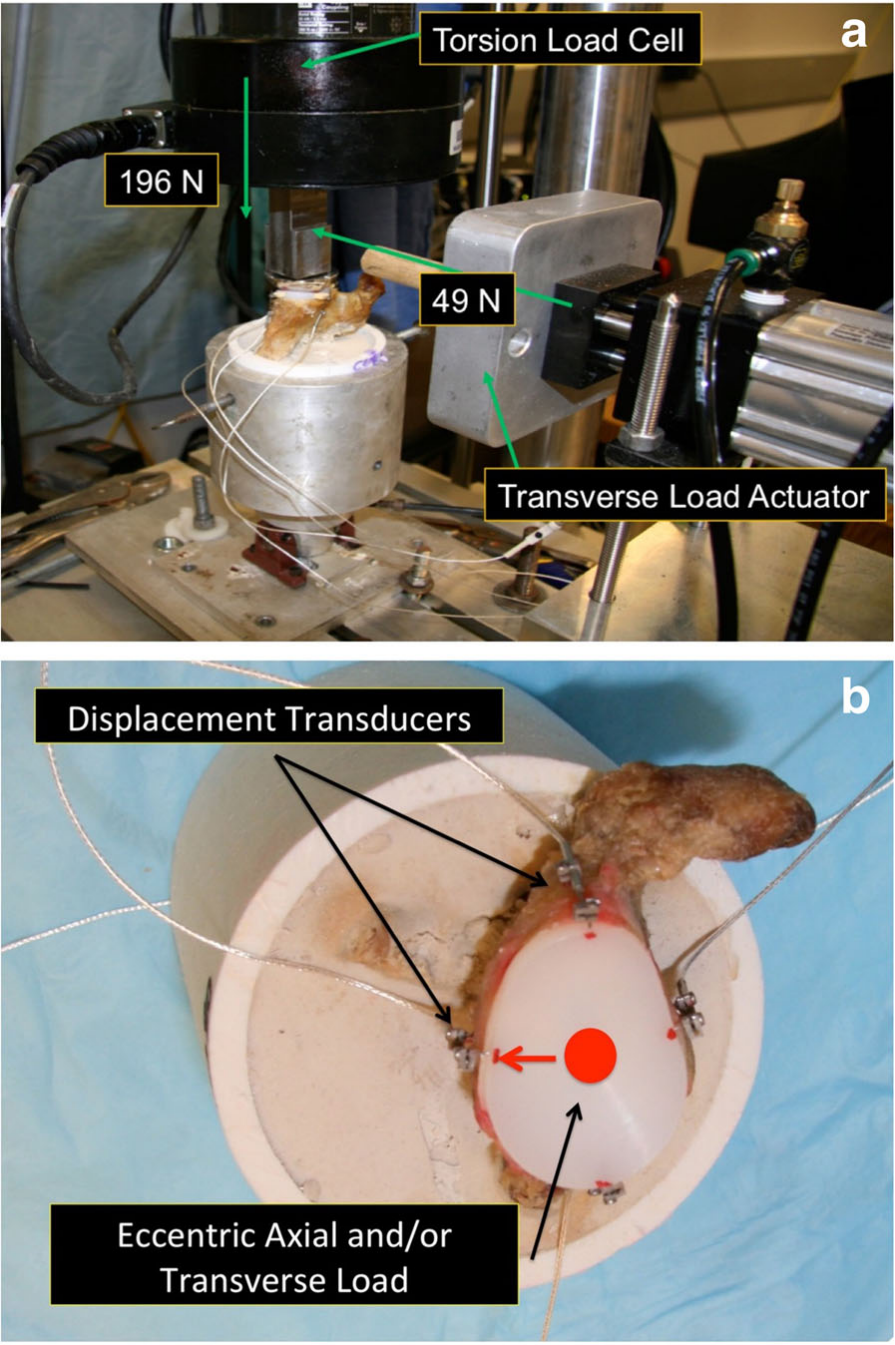
(*Left Photograph*) Biomechanical setup with an axial eccentric force of 196 N and transverse load of 49 N. (*Right Photograph*) Anterior, posterior, superior, and inferior position of the high-resolution differential variable reductance transducers for displacement measurement

Biomechanical testing of micro-instability under eccentric and axial loading was performed according to previously published methods [4]. In short, the scapula was fixed perpendicular to the MTS 858 Bionix II Servohydraulic testing system (MTS Systems Corp, Eden Prairie, MN, USA). The loading ball was replaced with a suitable humeral head in relation to the glenoid size. This configuration allows for load transfer as both components have been intended for the combined use, have a similar radius of curvature mismatch (Humeral Head (HH): 44/17 (22.6 mm), S-Glenoid radius of curvature (GRC): 29 mm ➔ Mismatch (MM) = 6.4; HH: 46/18 (23.7 mm), M-GRC: 30.5 mm ➔ MM = 6.8 mm; HH: 50/19 (25.9 mm), L-GRC: 32 mm ➔ MM = 6.1) and thus similar contact areas. In the first testing an axial eccentric force of 196 N was set on the pegged and keeled glenoid in all orientations: anteriorly (3‘o’clock), posteriorly (9‘o’clock), superiorly (12‘o´clock), and inferiorly (6‘o’clock). The eccentric point was defined as a 90% subluxation of each direction according to the biomechanical study of Anglin et al. [7] (Fig. 2) and starting position of each test series was checked visually. There was no randomized deflection. At each position the displacement of the glenoid component was determined with four different high-resolution differential variable reductance transducers (DVRT strain gauge, Microstrain, Burlington, VT, USA) placed anteriorly, posteriorly, superiorly, and inferiorly (Fig. 3). For the second testing protocol the starting point was defined as the center of the glenoid (0-position) with an axial load of 196 N. Additionally a transverse load of 49 N was applied in each direction: anteriorly (3‘o’clock), posteriorly (9‘o’clock), superiorly (12‘o’clock), and inferiorly (6‘o’clock). The eccentric load was slowly applied until 49 N was reached and was then held for 5 s. To make sure that an optimal transversal force transmission is provided a highly lubricated sled was used. This sled was mounted between the humeral head and the load cell of the MTS machine to allow both axial and transversal force transmission. The displacement of the glenoid component was measured as described above. All testing results refer to a single measurement. This loading protocol was selected according to previous studies simulating a load close to that predicted in vivo at 30 and 150° of abduction of the unweight arm (Fig. 3) [4, 8].

### Part 2: Radiolucency after a minimum of 24 months

Antero-posterior x-rays of 52 consecutive patients who underwent total shoulder arthroplasty with either a keeled or pegged glenoid component (same design and product used in biomechanical testing) of two shoulder specialized surgeons practices were evaluated after performing the biomechanical part. One surgeon only used keeled, the other one only used pegged. Patients were selected for this analysis if they underwent a primary total shoulder arthroplasty with one of the two implants used in biomechanical testing. Revision cases, arthroplasties performed with different components, and those without radiographs at a minimum of 2 years postoperatively were excluded. Radiolucency was graded according to the classification of Lazarus et al. [9] This classification grades the degree of radiolucency about pegged and keeled glenoid components starting from 0 (no radiolucency) to 5 (gross radiolucency). Two independent investigators (sports medicine trained orthopedic attending; sports medicine research fellow PGY3) graded all x-rays.

#### Statistical analysis

A power analysis (alpha value of 0.05 and power of 0.80) was performed based on a previous study and revealed a minimum of 15 specimens per group for biomechanical testing. Differences in age, bone mineral density (BMD), and glenoid surface between the two groups (pegged and keeled glenoid) were analyzed with a two sample T-test. Differences in the glenoid translation were analyzed with an Independent-Samples T-test. The Fisher’s Exact Test was used for analyzing differences in grading of radiolucency. Inter-rater-reliability for grading of radiolucency on x-ray was examined using the weighted Kappa statistic. The alpha level was 0.05 for all statistical tests. The analysis was conducted with SPSS version 22.0 (IBM, Armonk, NY).

## Results

### Part 1: biomechanics

#### Specimens

Before biomechanical testing, all specimens were scanned for bone mineral density (BMD) and the glenoid surface was identified. No statistically significant difference was found for BMD, glenoid surface, or age between the groups (*p* = 0.337, *p* = 0.991, *p* = 0.613) (Table 1).

**Table 1:** Mean and standard deviation of age, BMD (g/cm^-2^) and glenoid surface (mm^2^) from the tested specimens

Type of Glenoid	Number	Age, Mean ± Std. Deviation	BMD (g/cm^-2^), Mean ± Std. Deviation	Glenoid surface (mm^2^), Mean ± Std. Deviation
Pegged	15	62.9 ± 11.2	0.525 ± 0.164	705.85 ± 108.88
Keeled	15	60.9 ± 10.2	0.471 ± 0.135	706.34 ± 113.85
Alpha value		*p* = 0.337	*p* = 0.991	*p* = 0.613

#### Implantation of glenoid component (cement)

Because of the variation in glenoid anatomy we used 13 L-size (7 keeled, 6 pegged), 16 M-size (7 keeled, 9 pegged) and 1 S-size (1 keeled) glenoid component. Based on this variation, a subgroup analysis was performed to determine the amount of cement used in correlation to the size of the implant. Analysis determined that there was no statistically significant difference between the L-size keeled and pegged implants (*p* = 0.051). Additionally, there was no statistically significant difference between the M-size implant regarding the cement volume used (*p* = 0.224). Analysis of small size implants could not be performed because only 1 specimen required this sized implant. As the keeled implant has a greater anchor volume (1022.6 mm^3^) compared to the pegged (662.8 mm^3^) more bone had to be removed.

#### Glenoid displacement with eccentric axial load

**Fig. 4:**
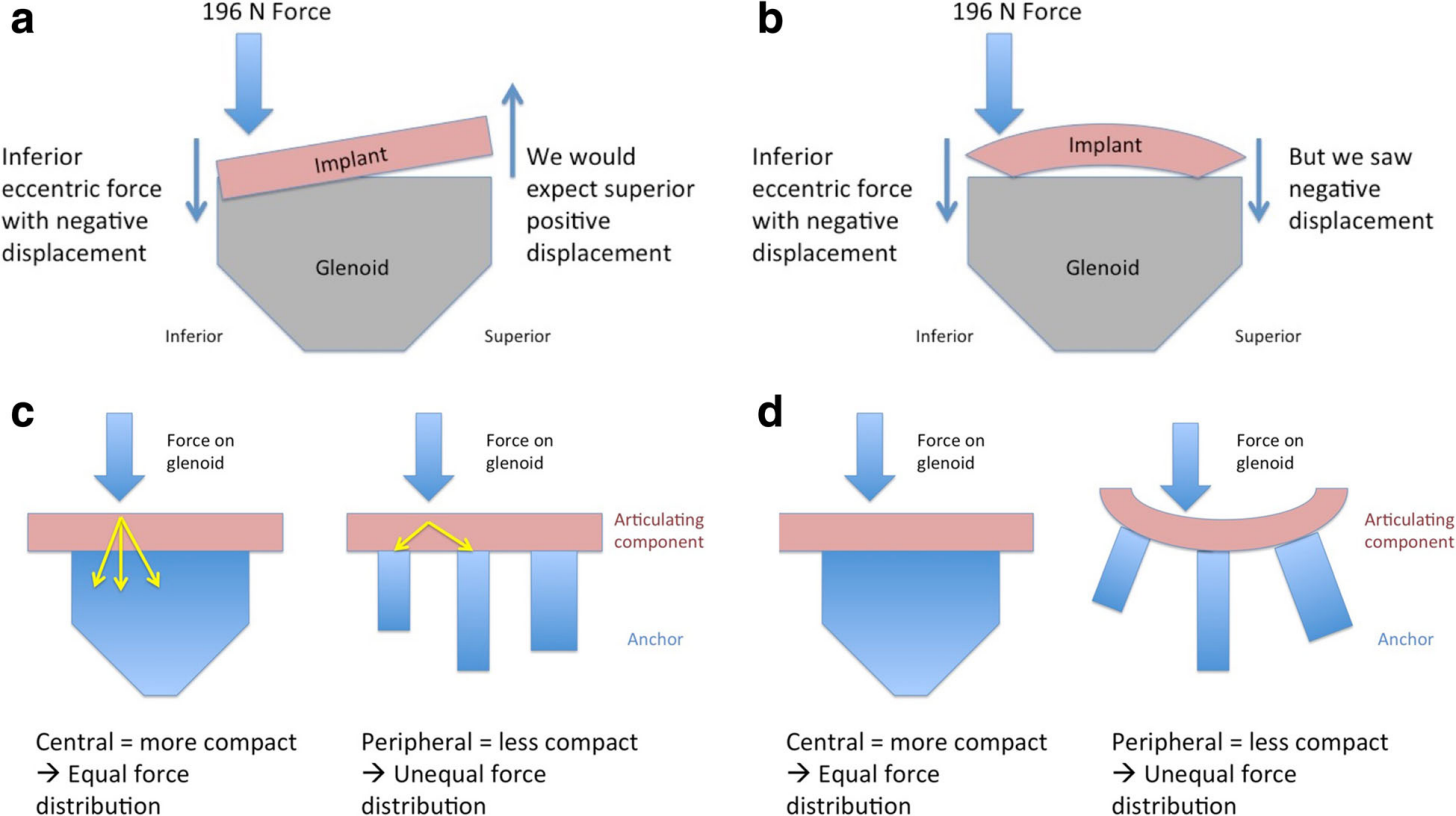
With an eccentric inferior force, we would expect a negative displacement for the component and a positive displacement in the superior aspect of the glenoid (**a**), but we saw a negative displacement (**b**) suggesting a deformation phenomenon. Furthermore, the anchor design would support this phenomenon, showing a more compact anchorage in the keeled component with equal force distribution (**c**) compared to the pegged glenoid with an unequal force distribution (**d**)

**Fig. 5:**
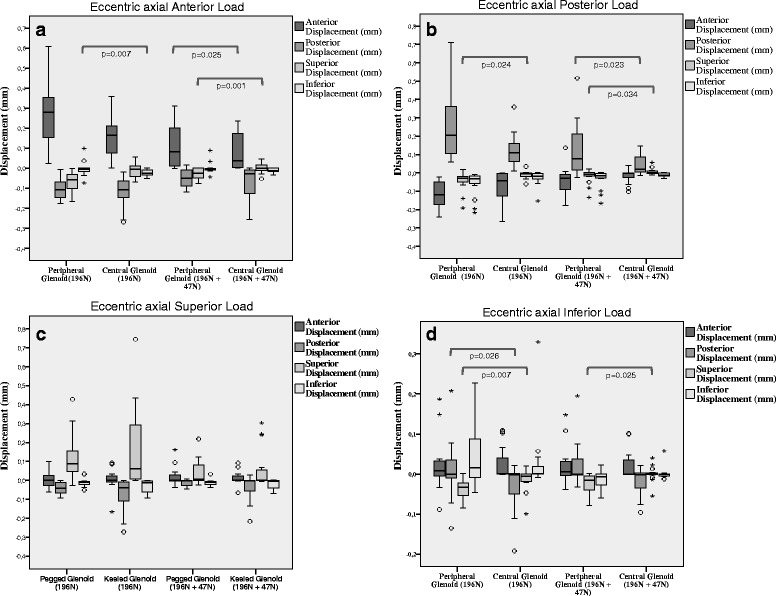
Comparison of displacements between single axial loading versus combined loading with an eccentric force. Boxplots represent means and standard deviation. The alpha level was 0.05 for all statistical tests and only significant results are reported

The glenoid displacement with eccentric axial load in the *anterior* direction showed a statistically significant difference between the pegged versus the keeled glenoid with more superior displacement in the pegged implant as compared to the keeled (*p* = 0.007). The eccentric *posterior* loading showed a difference with more superior displacement in the pegged implant as compared to the keeled (*p* = 0.024). The eccentric *superior* axial loading showed no statistically significant difference in displacement between the two glenoid components (*p* > 0.05). The eccentric *inferior* axial loading showed a difference with more superior and posterior displacement in the pegged glenoid (*p* = 0.007, *p* = 0.026). Also, a deformation phenomenon was observed in the pegged glenoid. Normally an interdependency displacement would be expected. For example, when applying an eccentric force to the inferior aspect of the glenoid component that results in positive inferior displacement (component goes down), we would expect a contrary negative displacement of the superior aspect of the glenoid component (component goes up). With the pegged component, there appeared to be a deformation phenomenon as the anterior and posterior displacement went in the same direction (negative displacement) while the superior and inferior displacement went in the other direction (positive displacement) (Fig. 4). Table 2 and Fig. 5 give an overview of the parameters resulting from eccentric axial loading.

**Table 2:** Displacement results after eccentric axial loading with a 196 N force for keeled and pegged glenoid components

Glenoid component	196 N axial force only	Displacement (Mean ± Std. Deviation in mm)			
		anterior	posterior	superior	inferior
Keeled Glenoid	anterior	0.08 ± 0.09	-0.07 ± 0.08	0.00 ± 0.03	-0.01 ± 0.01
	posterior	0.02 ± 0.04	0.04 ± 0.05	0.01 ± 0.02	-0.01 ± 0.01
	superior	0.01 ± 0.03	-0.03 ± 0.07	0.06 ± 0.11	-0.02 ± 0.02
	inferior	0.02 ± 0.04	-0.02 ± 0.04	0.00 ± 0.02	0.00 ± 0.02
Pegged Glenoid	anterior	0.11 ± 0.11	0.05 ± 0.04	0.03 ± 0.03^a^	0.00 ± 0.03
	posterior	0.05 ± 0.08	0.12 ± 0.15	0.02 ± 0.41^a^	0.03 ± 0.52
	superior	0.02 ± 0.05	-0.01 ± 0.02	0.04 ± 0.07	-0.01 ± 0.02
	inferior	0.02 ± 0.05	0.02 ± 0.06^a^	-0.02 ± 0.03^a^	-0.01 ± 0.02

(^a^ Statistical significant increased displacement compared to keeled glenoid, *p* > 0.05)

#### Glenoid displacement with eccentric axial and transverse loads

The eccentric axial loading in the anterior position resulted in a statistically significant difference with more superior and anterior displacement in the pegged glenoid as compared to the keeled (*p* = 0.001, *p* = 0.025). The eccentric axial loading in the posterior position resulted in a more superior and posterior displacement in the pegged glenoid as compared to the keeled (*p* = 0.023, *p* = 0.034). The eccentric axial loading in the superior position resulted in no difference in displacement between the two components (*p* > 0.05). The eccentric axial loading in the inferior position showed a statistically significant difference with more superior displacement in the pegged glenoid as compared to the keeled (*p* = 0.025). Table 3 and Fig. 5 give an overview of the parameters resulting from eccentric axial and transverse loading.

**Table 3:** Displacement results after eccentric axial loading with 196 N and 49 N transversal forces for keeled and pegged glenoid components

Glenoid component	196 N axial force + 49 N transversal force	Displacement (Mean ± Std. Deviation in mm)			
		anterior	posterior	superior	inferior
Keeled Glenoid	anterior	0.15 ± 0.11	-0.12 ± 0.08	0.01 ± 0.04	-0.02 ± 0.02
	posterior	-0.07 ± 0.08	-0.13 ± 0.09	-0.01 ± 0.02	-0.03 ± 0.04
	superior	0.00 ± 0.06	0.07 ± 0.09	0.17 ± 0.22	-0.03 ± 0.03
	inferior	0.03 ± 0.04	-0.03 ± 0.06	-0.02 ± 0.03	0.03 ± 0.08
Pegged Glenoid	anterior	0.27 ± 0.17^a^	0.10 ± 0.05	-0.07 ± 0.05^a^	-0.00 ± 0.04
	posterior	-0.12 ± 0.09	0.26 ± 0.19^a^	-0.04 ± 0.05^a^	-0.06 ± 0.07
	superior	0.00 ± 0.05	-0.04 ± 0.03	0.12 ± 0.12	-0.01 ± 0.02
	inferior	0.02 ± 0.07	0.01 ± 0.07	-0.04 ± 0.02^a^	0.04 ± 0.08

(^a^Statistical significant increased displacement compared to keeled glenoid, *p* > 0.05)

### Part 2: radiolucency after a minimum of 24 months

Fifty-two patients had radiographs with a minimum 2 years follow-up (Table 4). The mean follow up was 40.1 ± 11.7 months (peg: 41.6 ± 11.1, keel: 38.2 ± 12.5). There were 32 pegged and 24 keeled glenoids. The weighted Kappa for grading the x-rays was 0.72 indicating good inter-rater reliability [10]. Following the analysis of rater reliability all gradings were reviewed and disagreements only differed by one grade only. In these instances, the higher grade was selected for the statistical analysis.

**Table 4:** Demographics shown as ,mean and standard deviation of age (years) from postoperative radiological evaluation

Type of Glenoid	Number	Age, Mean ± Std. Deviation	Gender
Pegged	28	62.6 ± 9.1	19 male – 9 female
Keeled	24	69.3 ± 10.5	14 male – 10 female
All	52	65.7 ± 10.2	33 male – 19 female

**Fig. 6:**
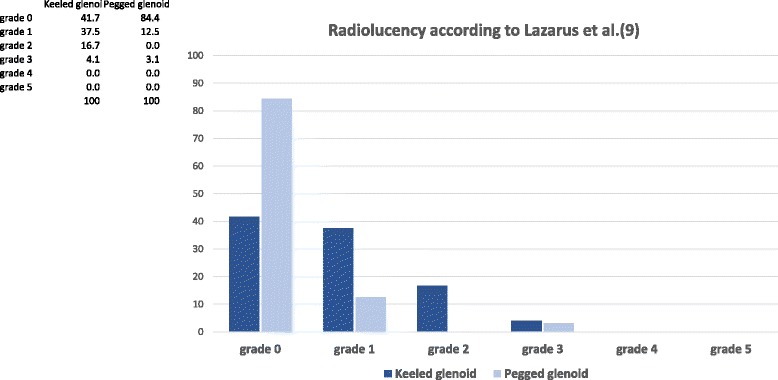
Illustration of radiolucency of keeled and pegged glenoid components according to the Lazarus classification

**Fig. 7:**
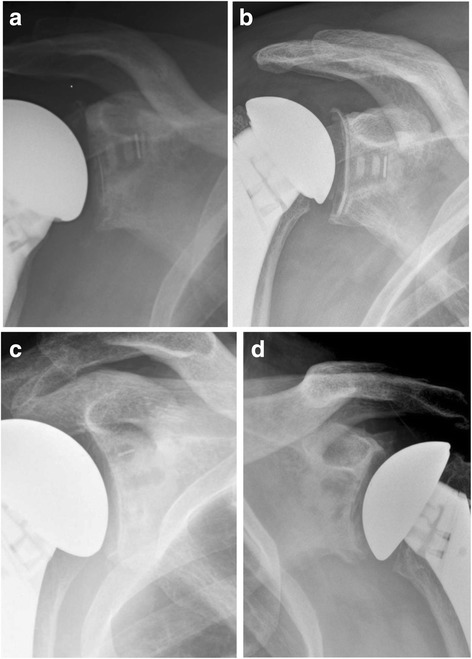
**a** Keeled glenoid component with a grade 1 and (**b**) with grade 2 radiolucency according to the Lazarus et al. [9] (**c**) Showing a pegged glenoid component with a grade 0 and (**d**) with a grade 1 radiolucency

Twenty-seven pegged glenoids showed a grade 0 (84.4%), 4 showed a grade 1 (12.5%) and 1 showed a grade 3 (3.1%). There were no radiolucency grades 2, 4 or 5 reported for pegged glenoids. Ten keeled glenoids showed a grade 0 (41.7%), 9 showed a grade 1 (37.5%), 4 showed a grade 2 (16.7%) and 1 showed a grade 3 (4.1%). However, no radiolucency grades 4 or 5 were reported for keeled glenoids (Fig. 6). All results were statistically significant with greater radiolucency for the keeled glenoid component (*p* = 0.001) (Fig. 7).

## Discussion

The goal of this study was to determine if the biomechanical performance of two glenoid components would correspond to the degree of radiolucency in patients at a minimum of 2 years following total shoulder arthroplasty. While the pegged component demonstrated more micro-motion during eccentric axial loading both with and without transverse force loads, the keeled component presented with a greater degree of radiolucency on postoperative x-rays. These findings provide support for both glenoid components from different perspectives and when taken together, highlight the need for well-constructed clinical studies to determine whether glenoid design influences outcome and patient satisfaction.

From a biomechanical perspective, the keeled component demonstrated less micro-motion in all orientations except for superior displacement. These findings are consistent or lower than those of Collins et al. [4] whose paper served as the basis of our testing protocol. The lack of a significant difference in superior displacement could be explained by the bony anatomy of the glenoid. Anglin et al. [11] reported a more stable bony socket in the superior glenoid as compared to the anterior, posterior, and inferior glenoid rim. Checroun et al. [12] reported that 71% of the 412 glenoids examined in their study displayed a pear-shaped form. These pear-shaped glenoids are described as having decreased width in the superior portion as compared to the inferior aspect [13]. Based on the differences in the dimension of the bony anatomy, complete coverage of the superior glenoid may not always be possible with an implant [14]. This may give the superior aspect of the glenoid component an advantage in terms of stability with eccentric loading. The anterior, posterior, and inferior aspects of the glenoid are well covered by the implant leaving less area to distribute eccentric loads in comparison to the superior aspect of the component where the lack of complete coverage results in bone above the implant thereby providing a larger surface area for eccentric force distribution.

The physical dimensions of the anchor on each component may have impacted stability. The single-anchor keeled glenoid is more compact and uniform in its design compared to the three-anchor pegged glenoid, which may influence the stability of the articulating surface of the component attached to the anchor (Fig. 4). This may explain the deformation of the pegged component during transverse loading. When loading the pegged glenoid with an inferior eccentric force, the component exhibited reciprocal displacements with motion in the anterior to posterior plane occurring in one direction and motion in superior to inferior plane occurring in opposite directions. For example, under an inferior eccentric force the anterior and posterior aspects of component both displaced anteriorly while the superior aspect displaced inferiorly and the inferior aspect displayed superiorly. This pattern of movement is considered a deformation phenomenon, indicating that the component itself had become deformed and may be explained by the connection between the articulating component and the anchoring component, which seems to be larger in the keeled glenoid. Additionally, the cement bone interface may have a role, but the impact cannot be answered with this biomechanical protocol [15].

The amount of bone removed when preparing the glenoid for implantation of the keeled or pegged components may have influenced the stability of the components. When investigating the different anchor sizes, we found a difference in volumes of the keeled (1022.6 mm^3^) versus the pegged (662.8 mm^3^) components indicating a greater amount of bone removal is required for the keeled glenoid. Additionally, the volume and weight of cement used during implantation showed no statistically significant differences between the two implants. These results may appear odd based on the assumption that a higher volume of implant would require more cement. A possible explanation of this finding may be that the preparation of the keeled component leads to the removal of more bone, which is typically cancellous bone. The cement for the keeled component is pressurized into the glenoid vault, which has less cancellous bone by virtue of the bone preparation, and therefore a better apposition to the cortical bone of the glenoid. With the pegged system, the bone removal is less, and therefore more of the cement is fixed within cancellous bone, which is less rigid and may deform under the testing conditions.

Over the last few decades the pegged and keeled glenoid components have been investigated regarding their ability to restore native glenoid function. Several biomechanical testing protocols and computer assisted finite element models were developed to determine which implant is more favorable. The proposed benefit of the pegged configuration is a more equal force distribution on the subjacent bone stock as demonstrated by finite element analysis [16, 17]. In contrast to the pegged confirmation, the keeled implant was designed to allow for easier surgical implantation and it may have also been designed as a keel due to manufacturing limitations when the first keeled glenoids were made. Lacroix et al. [18] compared the pegged versus keeled components and predicted that in 94% of pegged implants and 68% of keeled implants the cement has a greater than 95% probability of survival in normal bone. In bone of patients suffering from rheumatoid arthritis (RA), 86% of the pegged implants and 99% of the keeled implants were reported to have a greater than 95% probability of survival. Further, the results showed that bone stress is not substantially affected by the implant design, leading the authors to conclude that the pegged anchorage would be superior in the normal bone while keeled system would be superior for patients with rheumatoid arthritis or osteoporotic bone.

While the pegged component exhibited a greater amount of micro-motion during biomechanical testing, radiolucency was greater in patients with a keeled component. Edwards et al. [5] randomized 53 patients undergoing total shoulder arthroplasty (TSA) to either a pegged or keeled glenoid implant. At initial post-op examination, there was no difference in radiographic findings, but after a mean follow-up of 26 months the rate of glenoid radiolucency was significantly higher in patients with keeled glenoids (46%) as compared to patients with pegged glenoids (15%) (*p* = 0.003). Furthermore Gartsman et al. [19] reported an increased rate of radiolucency in keeled implants after 6 weeks with a rate of 39% and a significantly lower rate in pegged implants with a rate of 5% (*p* = 0.026). These findings are consistent with our results, showing more radiolucency for keeled compared to pegged glenoid components.

There is considerable debate regarding the relationship between radiographic findings and clinical failure. Long-term results from Torchia et al. [20] suggest a positive correlation. Walch et al. [21] reported that glenoid component failure is multifactorial and speculated that the preservation of glenoid bone stock is the most important factor in providing long-term resistance to the stress.

Other authors have not reported radiologic differences in patients with long-term follow up. Gazielly et al. [22] reported on long-term survival of keeled glenoid components in TSA with a mean follow-up of 8.5 years using a bone compaction and cement pressurization technique. These results were comparable to pegged components with radiological glenoid loosening of 15.5%. Throckmorton et al. [6] investigated 100 patients undergoing primary TSA with pegged and keeled glenoid components. At mean follow-up of 51.3 months 8% of pegged implants and 4% of keeled implants demonstrated radiographic lucency however there was no differences in clinical outcomes at intermediate-term follow-up (*p* = 0.74). Walch et al. [21] performed a multicenter study evaluating 518 TSA more than five years out from surgery. Radiographic loosening was present in 33% of the keeled components and was associated with three predominant patterns: 1) superior tilting, 2) subsidence and 3) posterior tilting. The authors proposed that the subchondral bone quality beneath the implant component is important to maintain the position of the glenoid over time.

The optimal method of long-term glenoid fixation has yet not been defined. Metal-back glenoids have the disadvantage of requiring more significant initial bone resection, risk of late metal on metal debris, increased overstuffing, and higher revision rates [23]. Boileau et al. [24] compared the cemented polyethylene glenoid to an unique uncemented metal-back glenoid component in a prospective, double blind randomized study. The results show more favorable outcomes with cemented polyethylene glenoids based on the significantly higher incidence of loosening with this unique metal-backed glenoid design as compared to polyethylene components. These findings are supported by the results from Fox et al. [25] who investigated 1337 patients with 1542 TSA using 6 types of glenoids (cemented, not cemented, polyethylene, keeled, pegged and metal-back). They concluded that optimal implant survival was achieved with the cemented all-polyethylene glenoid components with 15-year follow-up. Cemented all-polyethylene pegged or keeled glenoids are widely considered the optimal implants, as their outcomes are believed to be the most reliable [21, 25–27]. An additional advantage is the minimal amount of bone removal required for proper placement. Further research and development needs to be continued to determine the ideal shape of the glenoid and the method of fixation associated with the highest rate of radiographic and clinical stability.

There are limitations to this study. The in vitro nature of biomechanical evaluation can be a limiting factor in the translation of the findings to the in vivo conditions of the shoulder complex. This is particularly true for load distribution in shoulder replacement with its specific three-dimensional forces. Accurate replication of these forces in a cadaveric study is a challenge. Another limitation to this study is the fact that biomechanical testing has been performed with single loads, whereas occurrence of radiolucency is depended on repetitive cycles over the course of time in an actively remodeling system under ever changing loading scenarios. Thus, a correlation is hard and it is not clear if the cement bone interface has a significant contribution to this effect. In addition, the mean age of all cadaveric specimens was 61.9 ± 10.6 and therefore raises concerns about the quality of bone. However, shoulder replacement is commonly used in older patients and the bone mineral density of each cadaveric specimen was measured to ensure comparable results. Additionally, biomechanical testing with cadaveric specimens does not allow the effects of biological healing to be measured and, therefore, we are able to draw conclusions only for the primary stability of the joint at a time point immediately after implantation of the glenoid component. Furthermore, we did not evaluate the humeral head component position (imperfect or non-anatomical head replacement) on the radiographs as this may have affected the glenoid radiolucency and age difference in the compared groups may have influence on the radiographic findings, too (mean difference 6.7 years). Additionally, only two surgeons performed the arthroplasty. One only used keeled, the other one only used pegged components. This has a limitation by the surgeon and may provide a selection bias, but both surgeons are experienced and specialized shoulder surgeons.

## Conclusion

The biomechanical properties of glenoid components did not agree with the degree of radiolucency of two cemented basic glenoid designs for total shoulder arthroplasty. While the pegged component exhibited a greater amount of micro-motion during biomechanical testing, radiolucency was greater in patients with a keeled component. These findings provide support for both components from different perspectives and highlight the need for well-constructed studies to determine whether glenoid design has an effect on clinical outcome, because influences are multifactorial and biomechanical forces may not recreate forces seen in vivo.

## Abbreviations

BMD: Bone mineral density
DVRT: Differential variable reductance transducers
GRC: Glenoid radius of curvature
HH: Humeral head
MM: Mismatch
PGY3: Post graduate year 3
TSA: Total shoulder arthroplasty
